# Identification and validation of a signature based on macrophage cell marker genes to predict recurrent miscarriage by integrated analysis of single-cell and bulk RNA-sequencing

**DOI:** 10.3389/fimmu.2022.1053819

**Published:** 2022-11-11

**Authors:** Peiru Wei, Mingyou Dong, Yin Bi, Saiqiong Chen, Weiyu Huang, Ting Li, Bo Liu, Xiaoqian Fu, Yihua Yang

**Affiliations:** ^1^Guangxi Reproductive Medical Center, The First Affiliated Hospital of Guangxi Medical University, Nanning, China; ^2^Guangxi Key Laboratory of Immunology and Metabolism for Liver Diseases, Guangxi Medical University, Nanning, China; ^3^The Key Laboratory of Early Prevention and Treatment for Regional High Frequency Tumor, Guangxi Medical University, Ministry of Education, Nanning, China; ^4^The Key Laboratory of Molecular Pathology (For Hepatobiliary Diseases) of Guangxi, Affiliated Hospital of Youjiang Medical University for Nationalities, Baise, China; ^5^Department of Obstetrics and Gynecology, The Fourth Affiliated Hospital of Guangxi Medical University, Liuzhou, China

**Keywords:** recurrent miscarriage, single-cell RNA-sequencing, bulk RNA-sequencing, macrophage, maternal fetal interface

## Abstract

Recurrent miscarriage (RM) is a chronic, heterogeneous autoimmune disease that has serious social and personal consequences. No valid and reliable diagnostic markers or therapeutic targets for RM have been identified. Macrophages impact the innate immune system and can be used as diagnostic and prognostic markers for many diseases. We first collected 16 decidua and villi tissue samples from 5 normal patients and 3 RM patients for single-cell RNA sequencing data analysis and identified 1293 macrophage marker genes. We then screened a recurrent miscarriage cohort (GSE165004) for 186 macrophage-associated marker genes that were significantly differentially expressed between RM patients and the normal pregnancy endometrial tissues, and performed a functional enrichment analysis of differentially expressed genes. We then identified seven core genes (ACTR2, CD2AP, MBNL2, NCSTN, PUM1, RPN2, and TBC1D12) from the above differentially expressed gene group that are closely related to RM using the LASSO, Random Forest and SVM-RFE algorithms. We also used GSE26787 and our own collection of clinical specimens to further evaluate the diagnostic value of the target genes. A nomogram was constructed of the expression levels of these seven target genes to predict RM, and the ROC and calibration curves showed that our nomogram had a high diagnostic value for RM. These results suggest that ACTR2 and NCSTN may be potential targets for preventative RM treatments.

## Introduction

Recurrent miscarriage (RM), defined as the loss of two or more pregnancies, is a critical obstetric disease that has a prevalence of 1-2% (1). Possible causes of RM include antiphospholipid syndrome, genetic susceptibility, abnormal uterine structure, chronic endometritis and environmental factors. The etiology of 50% of RM cases is unknown (2). Maternal fetal interface immune status plays an important role during normal pregnancy by establishing maternal immune tolerance to the fetus and ensuring the dynamic balance of the immune microenvironment. Prior studies have shown that immune imbalance at the maternal fetal interface is related to the pathogenesis of RM, but the exact mechanism behind this relationship is still unclear (3).

Immune cells at the maternal fetal interface during early pregnancy include NK cells, macrophages, T cells, B cells and dendritic cells. Macrophages are the second most abundant subpopulation of immune cells in the decidua (4). Their unique phenotype and heterogeneity make them very important to the establishment and maintenance of pregnancy. Multiple studies have shown that RM is related to abnormalities in immune tolerance at the maternal fetal interface, thereby affecting embryo implantation and development and resulting in a miscarriage (5). Immunomodulators used clinically include glucocorticoids, cyclosporine A and hydroxychloroquine. However, the use of any of these drugs needs further confirmation due to imperfections in immune mechanism-related research (6, 7).

Macrophages are essential to the establishment and maintenance of a pregnancy as they are involved in a variety of processes, including vascular remodeling, immune tolerance, regulation of immunity by maternal meconium lymphocytes and the onset of labor (8–12). Macrophages are divided into 2 subtypes: pro-inflammatory M1 and anti-inflammatory M2. Numerous studies have shown that the ratio of M1/M2 macrophages in the decidual tissue of patients with unexplained RM is significantly increased compared with women with healthy pregnancies during the first trimester (13). Metaphase macrophages (dMΦ) account for 20-25% of the metaphase leukocyte population during early pregnancy (14) and are involved in vascular remodeling, inducing the apoptosis of damaged cells, removing apoptotic cell debris and eliminating invading pathogens (15). M1 macrophages can affect trophoblast invasion and migration, which can lead to pregnancy failure (16, 17). It has been reported that dMΦ may be polarized to the M2 subtype during early pregnancy to maintain that pregnancy (18), while macrophages polarized to the M1 subtype may promote RM (19). Some prior works have shown that adverse pregnancy outcomes can be reversed *via* macrophage polarization regulation, which is a potential target for drugs seeking to maintain pregnancies (20, 21). Further studies are needed to clarify the role of macrophages in maternal fetal interface immune tolerance.

The development of single-cell sequencing technology has permitted the study of the diversity of cell types, the specific molecular characteristics of the lineage and differentiation stage, and the functional interaction between cell types, which are required for the study of cell heterogeneity at the maternal fetal interface (8). Published literature on the maternal fetal interface has systematically described the relevant cell types (22–25). Single-cell sequencing has also played an irreplaceable role in the study of the etiology of RM. It has the advantage of searching for cell heterogeneity, some studies have indicated the immune heterogeneity of decidua and the potential pathogenic immune variations of RM (26–29). The present study established the cell map of the maternal fetal interface during early pregnancy, then proposed a prediction model for the diagnosis and treatment of unexplained RM by combining with the results of second-generation sequencing, permitting further exploration of the relationship between immune cells and unexplained RM.

## Materials and methods

### Data collection

In this study, single-cell RNA sequencing data were collected from decidual and villi samples from five women who underwent elective termination of normal pregnancies without a history of miscarriages and three women with RM at the First Affiliated Hospital of Guangxi Medical University. The inclusion criteria for RM participants were: (1) no fetal heart pulsation or fetal heartbeat stop using Doppler ultrasound at 7–9 weeks of gestation; and (2) a history of two or more failed pregnancies from an unknown cause. The exclusion criteria were: (1) fetal chromosomal karyotype abnormality of the villi; (2) patients with uterine anatomic disorders; and (3) patients with endocrine disorders (6 for single‐cell RNA sequencing). Decidual tissue and chorionic villi tissue from the same patient were collected separately, and a part of the chorionic villi tissue was sent for cytogenetic analysis. Informed consent was obtained from each patient before surgery. Ethical approval was obtained from the ethics committee of the First Affiliated Hospital of Guangxi Medical University. We also obtained 2 RM-related microarray datasets (GSE165004 and GSE26787) from the Gene Expression Omnibus (GEO) database. The GSE165004 (N=24; P=24) dataset was used to identify the core genes associated with RM, and the GSE26787 (N=5; P=5) dataset was used to further validate the expression profile of the signature genes. As these two public datasets were obtained from public databases, no ethical approval was required.

### Cell isolation

Decidual and placental tissue were washed in phosphate-buffered saline with 100 IU/mL penicillin/streptomycin and sheared into tiny pieces. Decidual tissue were digested with collagenase type IV (0.5 mg/ml, Invitrogen) for 30 min while the resultant villous tissue was enzymatically digested with EDTA (Sigma) while stirring at 37°C for 9 min. The disaggregated cell suspension was passed through 70 and 40 µm mesh sieves (Biologix), centrifuged, and resuspended in 3 mL of red blood cell lysis buffer (Invitrogen) for 3 min to exclude any remaining red blood cells. The pelleted decidual cells and placental cells were resuspended in PBS and used for single‐cell 3′‐cDNA library preparation followed by the 10× Genomics Chromium Single‐Cell 3′ reagent kit using the manufacturer’s instructions.

### Identification of macrophage marker genes *via* scRNA-seq analysis

Single‐cell libraries were sequenced with an Illumina NovaSeq 6000 using 150 nt paired‐end sequencing. The gene expression matrix was generated using CellRanger v3.1.1, and raw data were further processed with R (version 3.5.2). The quality control steps were as follows: (1) the number of identifiable genes in a single cell was between 500 and 7500, (2) the proportion of mitochondrial gene expression in a single cell was less than 20%, and (3) the total number of Unique Molecular Identifiers (UMIs) in a single cell was less than 50000. Data were normalized using the “normalizedata” function in the Seurat R package. Graph-based clustering was performed based on the gene expression profile of the cells using the “findclusters” function in Seurat (clustering resolution = 0.5, k-nearest neighbor = 10). After filtration, a total of 66078 cells were left for subsequent analysis. The CCA method was used to eliminate the batch effect of different samples. Graph-based clustering was performed according to the gene expression profile of cells using the “findclusters” function in Seurat (clustering resolution = 0.5, k-nearest neighbor = 10). t-distributed random neighbor embedding (t-SNE) was then performed using the “Run tsne” function. Cell clusterings was demonstrated using t-SNE-1 and t-SNE-2. Differentially expressed genes (DEGs) were calculated for each cluster using the Wilcoxon-Mann-Whitney test using the “FindAllMarkers” function in the Seurat package. To identify the marker genes for each cluster, we used cut-off thresholds adjusted for p-values < 0.01 and |log2 (fold change) > 1. We used the “SingleR” R package for automated annotation of cell types to annotate the cell subgroups.

### Identification of macrophage marker genes associated with RM

By using the limma R package (30), the GSE165004 dataset (containing 24 healthy individuals and 24 RM patients) was used to screen genes that were significantly differentially expressed between the RM group and the normal group (Screening label: False Discovery Rate criterion (FDR) < 0.05). All differentially expressed genes were intersected with macrophage marker genes to identify the macrophage markers associated with RM.

### Functional enrichment analysis of differentially expressed genes

The ClusterProfiler R package (31) was used to perform a biological functional enrichment analysis of differentially expressed genes with Gene Ontology (GO) and the Kyoto Encyclopedia of Genes and Genomes (KEGG). FDR was used to perform multiple test corrections with a threshold set at <0.05. GO categories were analyzed for biological processes only (BP).

### Selection of feature genes

Three machine learning algorithms: LASSO, Random Forest and SVM-RFE (32), were used to screen signature genes. Differentially expressed macrophage marker genes were initially assessed by least absolute shrinkage and selection operator (LASSO) Cox proportional hazards regression using the “glmnet” R package. The value of the penalty parameter (λ) corresponding with the lowest partial likelihood deviance was used to select the best model *via* 10-fold cross-validation (33). Genes with non-zero beta coefficients were retained. A random forest algorithm was used to rank the importance of marker genes associated with RM, and genes with an importance greater than 0.25 were used for subsequent analyses. SVM-RFE was used to further screen for signature genes, with the top 12 genes by average ranking retained for subsequent analyses. Genes identified using LASSO, random forest and SVM-RFE were intersected to obtain our signature genes. ROC curves were used to assess the efficacy of those signature genes to distinguish the RM samples. We also tested the diagnostic value of the signature genes in GSE26787.

### Construction of a protein-protein interaction network

GeneMANIA (http://www.genemania.org) is a website for building protein-protein interaction (PPI) networks (34), which can generate gene function predictions and locate genes with comparable effects. Physical interaction, co-expression, co-localization, gene enrichment analysis, genetic interaction and locus prediction are some of the bioinformatics methods used by network integration algorithms. In this study, GeneMANIA was used to analyze the PPI networks of our signature genes. The ClusterProfiler R package (31) was used to perform a GO and KEGG enrichment analysis of interacting proteins, FDR<0.05 was used to screen for significantly enriched pathways, and the top 10 most significant signaling pathways were presented.

### RNA extraction and quantitative reverse‐transcriptase polymerase chain reaction analyses

Total RNA was extracted from fresh-frozen decidual samples obtained from the RM (n = 5) and normal (n = 5) groups using TRIzol (Takara, Japan) according to the manufacturer’s protocol. The RNA was then reverse-transcribed into cDNA using the Prime Script RT reagent Kit (Takara, Japan). The real‐time PCR system used RR420A TB Green™ Premix Ex Taq™ (Tli RNaseH Plus) (Takara, Japan). The Primer-BLAST online tool (https://www.ncbi.nlm.nih.gov/tools/primer-blast/index.cgi?LINK_LOC=BlastHome) was used to design primers and assess primer specificity. Primer lengths were set between 20 bp and 25 bp, GAPDH was selected as an internal reference gene, the total volume of fluorescent quantitative PCR amplification was 20ul, the primers used in this study are listed in **Table 1**. Relative fold changes in gene expression were calculated using the 2−ΔΔCt method and normalized with respective controls.

**Table 1:** Primers used in this study.

Primer Name	Prime (5’to3’)	Length (bp)
ACTR2-F	AGTTGGGTTCAGGGAAATGGG	21
ACTR2-R	CAAGGGACAAACGATAAATGCTC	23
CD2AP-F	AAAAGAAGAAGACAGTGCCAACC	23
CD2AP-R	AATGGAGTCAGGAAAGCAGTTGT	23
MBNL2-F	TCATACCCCACCAAACAAAGTC	22
MBNL2-R	GAAGTCTGGCAAAATCTAGGCAC	23
NCSTN-F	TGGGCAATGGTTTGGCTTAT	20
NCSTN-R	CAGGTGGCAGTGCTGATGAC	20
PUM1-F	TGAAGAACGGTGTTGACTTAGGG	23
PUM1-R	TGGTTGCTGGTTGGATTTGC	20
RPN2-F	CGAGCCAGACAACAAGAACG	20
RPN2-R	CCTCAGGGAACTTGATGACCAC	22
TBC1D12-F	GCCCTCGTCTACCTCACTATCC	22
TBC1D12-R	CATTCCTCAAAGTATTTCACCTCCA	25
GAPDH-F	GGAGTCCACTGGCGTCTTCA	20
GAPDH-R	GTCATGAGTCCTTCCACGATACC	23

### Gene set enrichment analysis

In order to analyze the signaling pathways impacted by the signature genes, single-gene GSEA enrichment analysis was performed on the signature genes (35). We downloaded the “c2.cp.kegg.v11.0.symbols” gene set from the MSigDB database (http://software.broadinstitute.org/gsea/msigdb) (36) as a reference gene set for GSEA analysis. To achieve a normalized enrichment score for each analysis, gene set permutations were set at 1,000 times and FDR < 0.05 was considered a significantly enriched signaling pathway.

### Modeling and testing of a RM diagnostic nomogram

We created a nomogram for the diagnosis of RM using the rms R package. A risk score was calculated based on the expression of individual core genes, with the total risk score defined as the sum of all individual gene risk scores. The diagnostic value of the nomogram for RM was assessed using decision tree, calibration and ROC curves.

### Gene set variation analysis

GSVA is an unsupervised and non-parametric gene set enrichment method that permits the use of gene expression profiles to assess associations between biological pathways and gene features (37). We downloaded 50 hallmark gene sets from the MSigDB database (http://software.broadinstitute.org/gsea/msigdb) (36) to serve as reference gene sets. We used the ssGSEA function in the GSVA package to calculate the GSVA score for each gene set in different samples. The Limma package was then used to compare differences in the GSVA scores of different gene sets between the normal and disease groups.

### Analysis of immune cell infiltration and immune-related pathways

Sixteen immune cells and 13 immune-related pathway gene sets were obtained from the annex of the reference (38). Using these 29 gene sets and gene expression matrices as input files, single sample gene set enrichment analysis (ssGSEA) was performed on all samples using “gsva” R (39). Infiltration scores for 16 immune cells and the activity of 13 immune-related pathways were calculated for all samples.

### Tumor immune single-cell hub database analysis

The TISCH database includes 2045746 cells from 79 databases from tumor patients and healthy donors. These datasets were processed uniformly to permit the analysis of immune cell components at the single cell and annotated cluster levels. This work used datasets from TISCH to characterize the expression distribution of genes within different cell types at the single-cell level.

### Prediction of the drug sensitivity of target genes

The Drug Signature Database (DSigDB) was used to screen for key drugs that can target specific genes. A publicly available web database obtained from the Enrichr web server (https://maayanlab.cloud/Enrichr/), the DSigDB database provides association information for drugs and their target genes and is used to perform gene set enrichment analysis (GSEA). We identified drug candidates for the possible treatment of RM based on a statistical threshold of p-value < 0.05 and drug targets > = 2.

### Statistical analysis

Categorical variables were compared between different risk groups using the Wilcoxon t-test. Univariate and multivariate Cox regression analyses were used to calculate the prognostic value of macrophage CMGS and different clinicopathologic features. p<0.05 was defined as significant for all calculations. Benjamini-Hochberg adjusted p-values for multiple testing were calculated using the R function “p.adjust”. R software version 4.1.0 (http://www.R-project.org) was used for data analysis and graph generation.

## Results

### Single‐cell atlas at the maternal fetal interface in normal and RM samples

**Figure 1:**
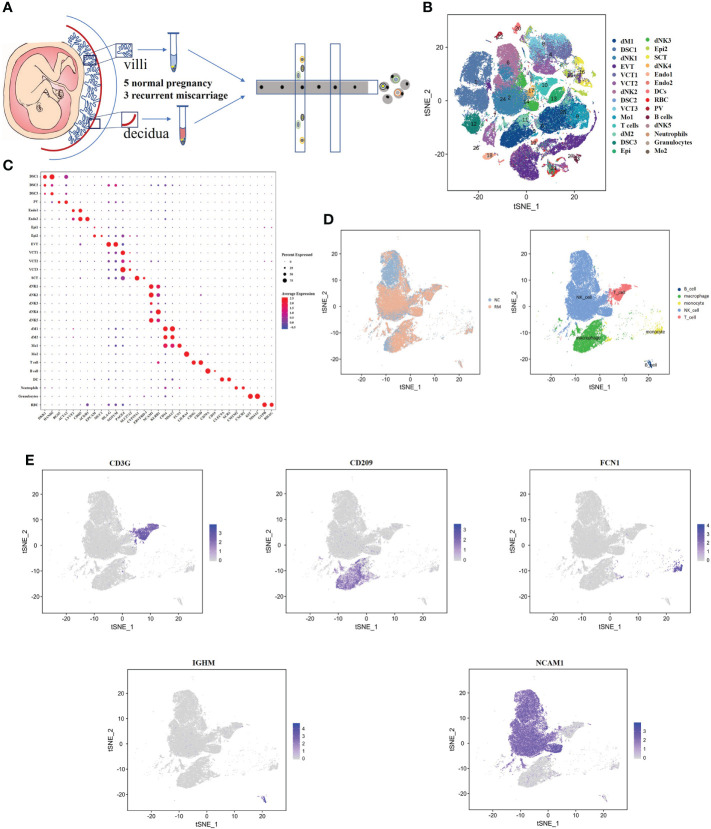
Single-cell RNA-sequencing analysis identifies maternal fetal interface marker genes **(A)** Flowchart depicting the overall design of the single-cell RNA-sequencing analysis. **(B)** t-SNE plot of 112528 cells from five normal samples and three RM samples. dM, decidual macrophages; dNK, decidual NK cells; DSC, decidual stromal cells; EVT, extravillous trophoblast; VCT, villous cytotrophoblast; Mo, Monocytes; Endo, endothelial cells; SCT, syncytiotrophoblast; DC, dendritic cells; RBC, red blood cells; PV, perivascular cells; Epi, epithelial glandular cells; F, fibroblasts. **(C)** Dotplot map showing the expression of classical cell type‐specific marker genes in each cluster. **(D)** t-SNE plot of the five main immune cell types. **(E)** t-SNE plot showing the expression of the marker genes of the five cell types noted above.

We obtained 16 human first-trimester decidual and villi samples from 5 normal patients and 3 RM patients using 10× Genomics, with decidual and villi samples collected from the same patient (**Figure 1A**). Following computational quality control and graph‐based clustering using the Seurat package (40), 112528 high-quality cells were subjected to further analysis. These cells consisted of 36219 cells from normal decidua and 25582 cells from RM decidua, 25303 cells from normal villi and 25424 cells from RM villi. After unsupervised graph-based analysis using SingleR software (41), we automatically annotated the cell clusters and identified 15 cell types (containing 28 subclusters) assigned on the basis of known marker genes and literature evidence, and cells in cluster 0 and 11 were defined as macrophage cells (23, 25) (**Figures 1B, C**; **Supplementary Table 1**). We then extracted dNK cell, T cell, macrophage, monocyte and B cell populations related to the immune mechanism of RM and regrouped them based on the expression of known marker genes for further analysis (**Figures 1D, E**).

### Identification and functional enrichment analysis of differentially expressed macrophage-associated genes

**Figure 2:**
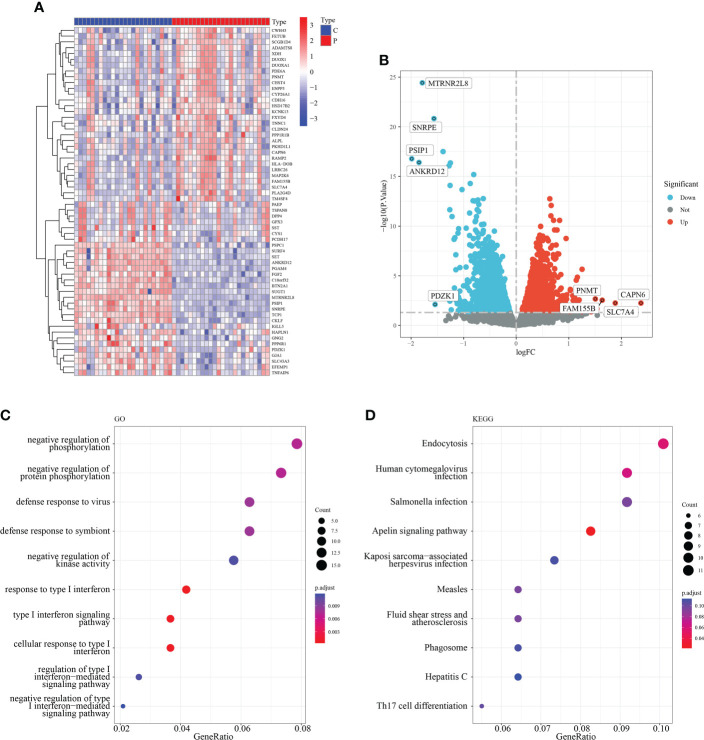
Identification of macrophage-related differentially expressed genes. **(A)** Heat map of the top 50 DEG. **(B)** Volcano plot of DEG. **(C)** GO enrichment analysis of DEG. **(D)** KEGG enrichment analysis of DEG.

To investigate the role of macrophage-associated genes in the pathogenesis of RM, we used the expression profiles of 24 normal and 24 recurrent miscarriage specimens from the GSE179996 cohort for subsequent analysis. A total of 1,293 macrophage-associated marker genes were obtained *via* the single-cell sequencing screening of 1,384 macrophage-associated genes, which were intersected with genes from the GSE179996 expression profile. Differential expression analysis of the GSE179996 dataset revealed that 90 macrophage marker genes were significantly down-regulated and 96 macrophage marker genes were significantly up-regulated in the disease group (**Figures 2A, B**). Detailed information is listed in **Supplementary Table 2**. GO enrichment analysis of these differentially expressed macrophage-associated genes revealed that they impacted negative regulation of protein phosphorylation, the type I interferon signaling pathway and the immune response to viruses (**Figure 2C**). KEGG enrichment analysis of these differentially expressed genes revealed that the main signaling pathways involved were endocytosis, human cytomegalovirus infection, the Apelin signaling pathway and Th17 cell differentiation (**Figure 2D**).

### Screening for signature genes using the LASSO, random forest and SVM-RFE algorithms

**Figure 3:**
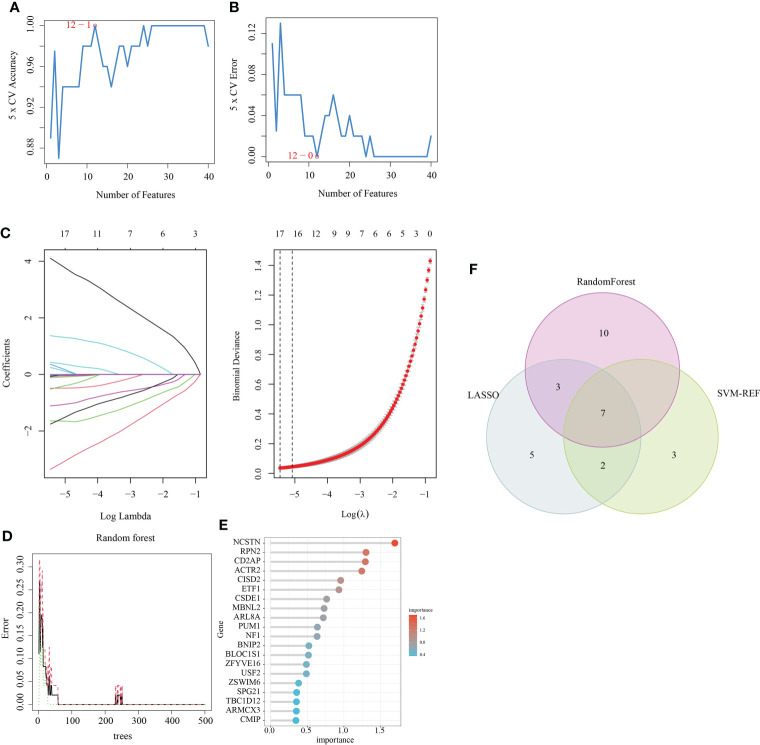
Feature gene selection. **(A, B)** Biomarker signature gene expression validation *via* support vector machine recursive feature elimination (SVM–RFE) algorithm selection. **(C)** Adjustment of feature selection in the minimum absolute shrinkage and selection operator model (LASSO). **(D)** random Forest error rate versus the number of classification trees. **(E)** The top 20 relatively important genes. **(F)** Three algorithmic Venn diagram screening genes.

Three algorithms (LASSO, Random Forest and SVM-RFE) were used to screen for the core signature genes associated with RM progression. With respect to SVM-RFE, classifier error was minimized when the number of features was 12, containing CD2AP, NCSTN, APPL1, ACTR2, PTMS, TBC1D12, ATF6, MBNL2, RPN2, MTMR6, MAFG and PUM1 (**Figures 3A, B**). With respect to the LASSO algorithm, after ten cross-validations the best lambda was 0.004. The value of penalty parameter (λ) corresponding with the lowest partial likelihood deviance was used to select 17 signature genes: ACTR2, APPL1, ARL8A, ARMCX3, CD2AP, CSDE1, ITGB2, MBNL2, NCSTN, OAS1, PML, PTMS, PUM1, RPN2, SLC43A3, SPPL2A and TBC1D12 (**Figure 3C**). Twenty feature genes with relative importance >0.345 were identified using the random forest algorithm, including NCSTN, RPN2, CD2AP, ACTR2, CISD2, ETF1, CSDE1, MBNL2, ARL8A, PUM1, NF1, BNIP2, BLOC1S1, ZFYVE16, USF2, ZSWIM6 SPG21, TBC1D12, ARMCX3 and CMIP (**Figures 3D, E**). Seven shared feature genes from the LASSO, Random Forest and SVM-RFE algorithms were identified: ACTR2, CD2AP, MBNL2, NCSTN, PUM1, RPN2 and TBC1D12 (**Figure 3F**); The functional annotation of these genes in the NCBI database is shown in **Table 2**.

**Table 2:** Functional annotation of feature genes.

Primer Name	Ensembl_Gene_id	Functional annotation
ACTR2	ENSG00000138071	Actin related protein 2
CD2AP	ENSG00000198087	CD2 associated protein
MBNL2	ENSG00000139793	Muscleblind like splicing regulator 2
NCSTN	ENSG00000162736	Nicastrin
PUM1	ENSG00000134644	Pumilio RNA binding family member 1
RPN2	ENSG00000118705	Ribophorin II
TBC1D12	ENSG00000108239	TBC1 domain family member 12

### Diagnostic efficacy and external validation of signature genes in RM

**Figure 4:**
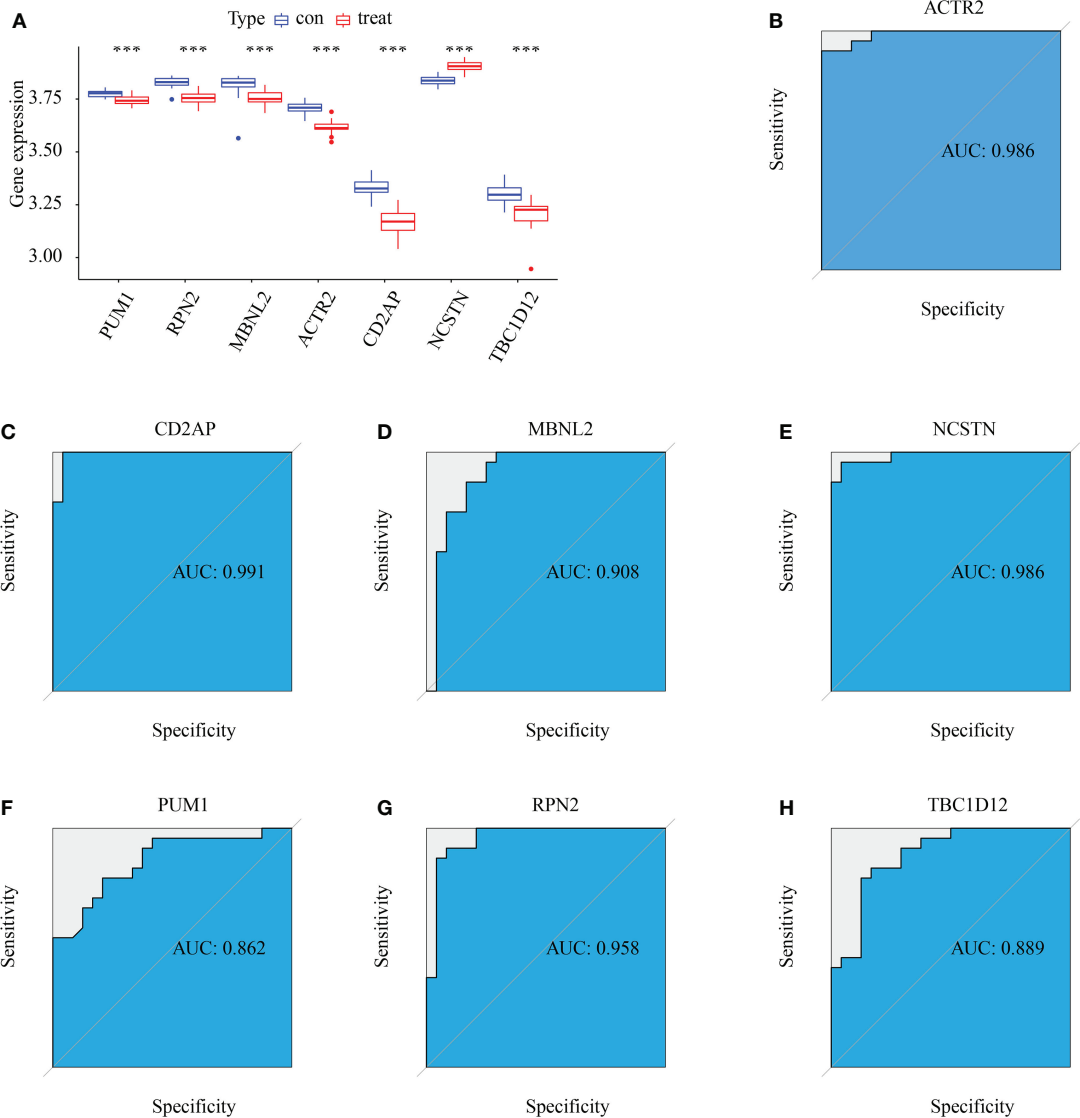
Diagnostic efficacy of the target genes in the prediction of RM. **(A)** Box plots showing the mRNA expression of the target genes in stable and unstable atherosclerotic plaque specimens in the GSE165004 dataset. **(B–H)** ROC curves estimating the diagnostic performance of the target genes **(B)** ACTR2, **(C)** CD2AP, **(D)** MBNL2, **(E)** NCSTN, **(F)** PUM1, **(G)** RPN2 and **(H)** TBC1D12 in the prediction of atherosclerotic plaque progression in the combined GSE165004 datasets. ***p < 0.001.

**Figure 5:**
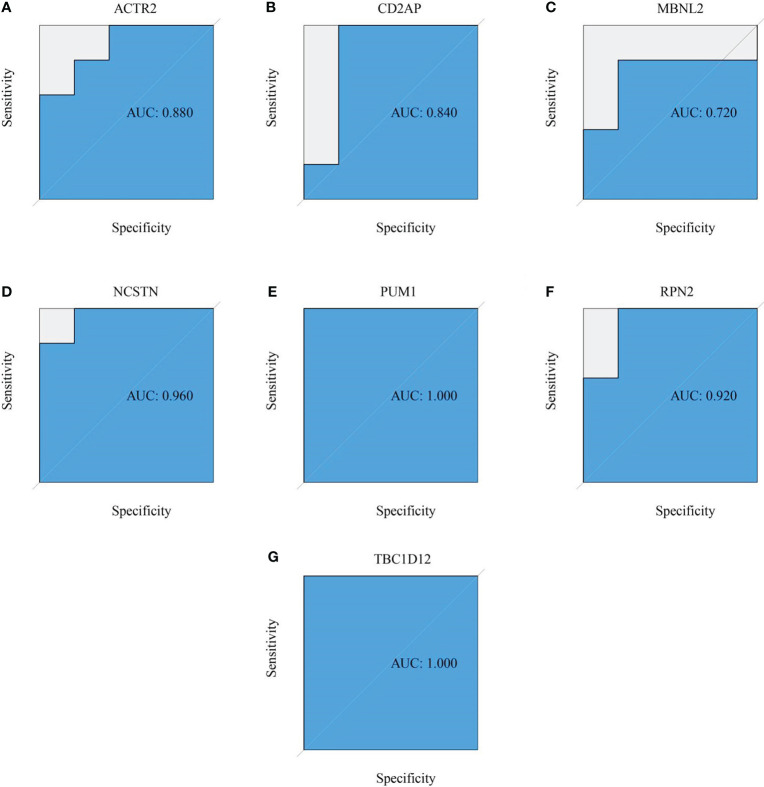
External verification of the RM predictive value of the target genes. **(A–G)** ROC curves estimating the diagnostic performance of the target genes **(A)** ACTR2, **(B)** CD2AP, **(C)** MBNL2, **(D)** NCSTN, **(E)** PUM1, **(F)** RPN2 and **(G)** TBC1D12 in the prediction of a RM in the GSE26787 datasets.

Six signature genes (ACTR2, CD2AP, MBNL2, PUM1, RPN2 and TBC1D12) were expressed at significantly lower levels in RM samples, while NCSTN had a significantly higher expression level (**Figure 4A**). The estimated diagnostic performance of the seven signature genes in the prediction of RM in the GSE179996 cohort using AUC values, which were 0.986 for ACTR2 (**Figure 4B**), 0.990 for CD2AP (**Figure 4C**), 0.908 for MBNL2 (**Figure 4D**), 0.986 for NCSTN (**Figure 4E**), 0.862 for PUM1 (**Figure 4F**), 0.958 for RPN2 (**Figure 4G**) and 0.889 for TBC1D12 (**Figure 4H**). We selected the GSE26787 dataset to externally validate the diagnostic value of these seven signature genes in the progression of recurrent miscarriage, which produced AUC values of 0.880 for ACTR2 (**Figure 5A**), 0.840 for CD2AP (**Figure 5B**), 0.720 for MBNL2 (**Figure 5C**), 0.960 for NCSTN 0.960 (**Figure 5D**), 1.000 for PUM1 (**Figure 5E**), 0.920 for RPN2 (**Figure 5F**) and 1.000 for TBC1D12 (**Figure 5G**).

### Experimental validation of signature gene expression

**Figure 6:**
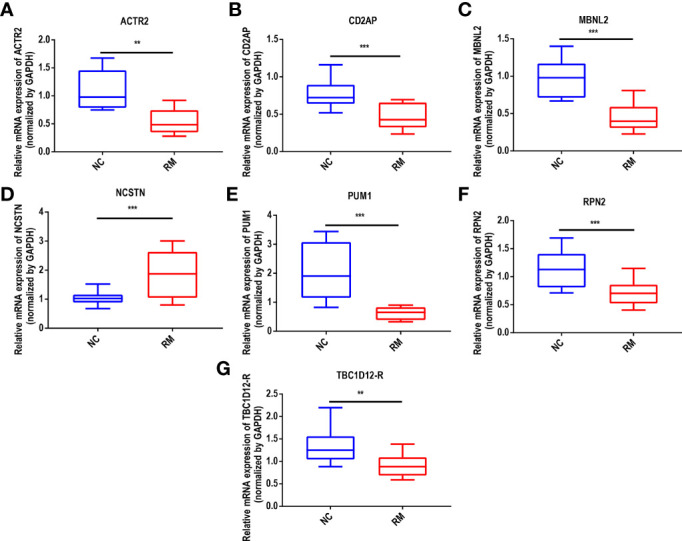
Expression validation of ACTR2 between normal and RM tissues. **(A)** ACTR2, **(B)** CD2AP, **(C)** MBNL2, **(D)** NCSTN, **(E)** PUM1, **(F)** RPN2 and **(G)** TBC1D12, "**p < 0.01, ***p < 0.001.

To validate the expression of the signature genes implicated RM, 10 healthy human meconium samples and 10 meconium samples from recurrent miscarriages were collected for clinical specimen validation. The expression patterns of the 7 signature genes were consistent between the GSE165004 dataset and the clinical specimens, further suggesting that they had good diagnostic value in the prediction of RM progression (**Figure 6**).

### Signaling pathways involved in signature genes

**Figure 7:**
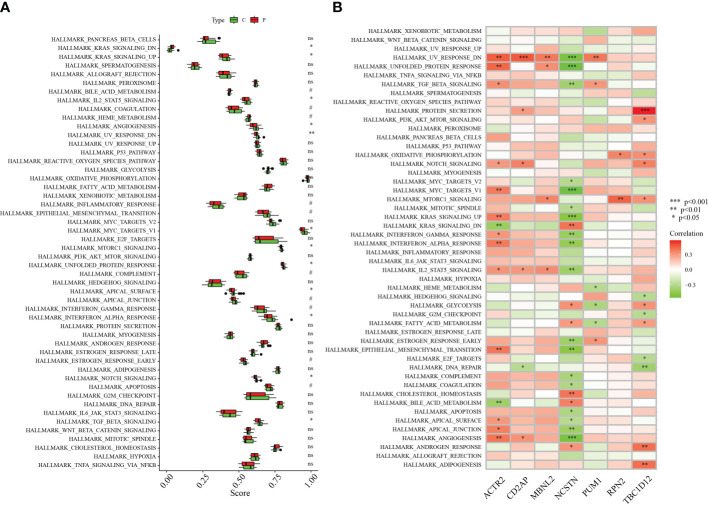
Correlation between hub genes and the 50 HALLMARK signaling pathways. **(A)** Comparison of the 50 HALLMARK signaling pathways between the RA group and healthy controls. **(B)** Correlation between the target genes and the 50 HALLMARK signaling pathways. *p < 0.05, **p < 0.01, ***p < 0.001 0.05. 0.05 < p < 0.2. NS, no significance.

The differences between RM patients and healthy controls within 50 HALLMARK signaling pathways were further investigated using ssGSEA. Two HALLMARK signaling pathways were significantly up-regulated in RM patients: KRAS_SIGNALING_DN and BILE_ACID_METABOLISM (**Figure 7A**). Pathways significantly down-regulated in RM patients included: KRAS_SIGNALING_UP, ANGIOGENESIS, UV_RESPONSE_DN, MYC_TARGETS_V1, MTORC1_SIGNALING, UNFOLDED_PROTEIN_RESPONSE, APICAL_SURFACE, INTERFERON_ALPHA_RESPONSE, NOTCH_SIGNALING and TGF_BETA_SIGNALING **(** **Figure 7A**). We also analysed the correlations of seven signature genes with 50 HALLMARK signaling pathways. ACTR2 was associated with a number of genes, including NOTCH_SIGNALING, KRAS_SIGNALING_UP, INTERFERON_GAMMA_RESPONSE and ANGIOGENESIS. Several HALLMARK signaling pathways including ANGIOGENESIS were significantly positively correlated with these genes (**Figure 7B**). In contrast, NCSTN was significantly negatively correlated with multiple HALLMARK signaling pathways including UV_RESPONSE_DN, UNFOLDED_PROTEIN_RESPONSE, TGF_BETA_SIGNALING, KRAS_SIGNALING_UP and INTERFERON_GAMMA_RESPONSE (**Figure 7B**).

**Figure 8:**
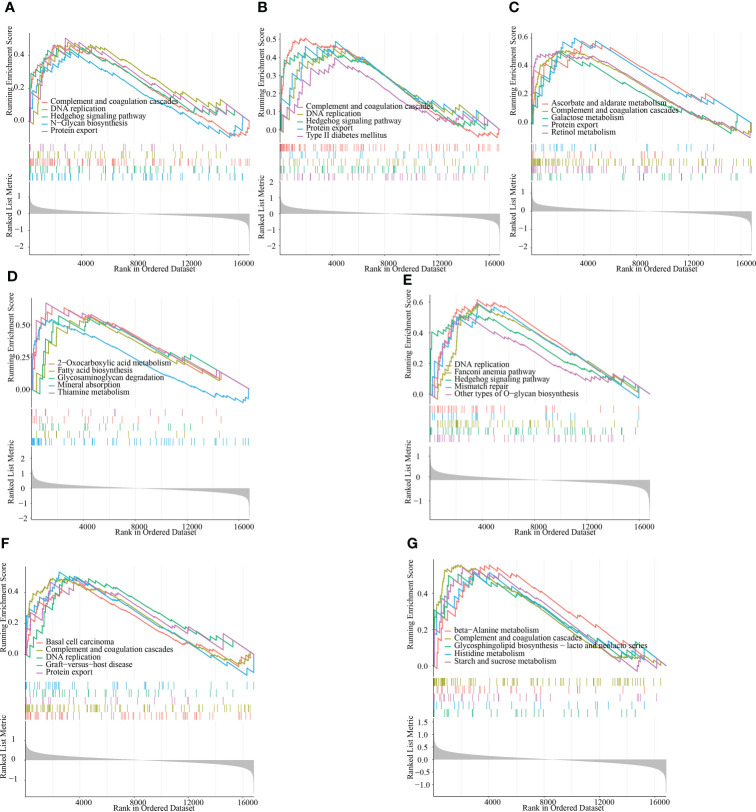
GSEA identifies the signaling pathways that are impacted by the target genes. **(A–G)** The main signaling pathways that are significantly enriched by the high expression of the target genes in the GSE165004 dataset. **(A)** ACTR2, **(B)** CD2AP, **(C)** MBNL2, **(D)** NCSTN, **(E)** PUM1, **(F)** RPN2 and **(G)** TBC1D12.

To provide more clarity on the impact of the signature genes on the progression of RM, we performed single-gene GSEA enrichment analysis. Both ACTR2 and CD2AP are involved in the complement and coagulation cascades, DNA replication, the Hedgehog signaling pathway and the protein export signaling pathways. The signaling pathways impacted by RPN2 mainly include basal cell carcinoma, the complement and coagulation cascades, DNA replication and protein export (**Figures 8A, B**). MBNL2 is involved in the following signaling pathways: ascorbate and aldarate metabolism, the complement and coagulation cascades, DNA replication and protein export (**Figure 8C**). NCSTN is involved in the following signaling pathways: fatty acid biosynthesis, glycosaminoglycan degradation and mineral absorption (**Figure 8D**). PUM1 is involved in the following signaling pathways: DNA replication, the Hedgehog signaling pathway and mismatch repair (**Figure 8E**). RPN2 is involved in the following signaling pathway: Basal cell carcinoma, complement and coagulation cascades, DNA replication, and protein export (**Figure 8F**); The main signaling pathways impacted by TBC1D12 include beta-alanine metabolism, the complement and coagulation cascades, the H coagulation cascades and histidine metabolism (**Figure 8G**).

### Characterized gene interaction network analysis

**Figure 9:**
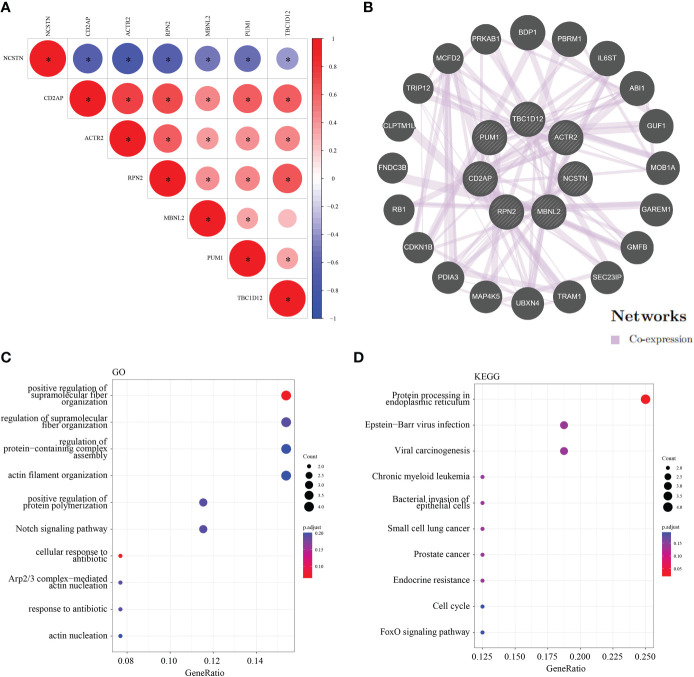
Co-expression and interaction analysis of the target genes. **(A)** Target gene co–expression network in GSE165004. **(B)** Target gene co–expression network in GeneMANIA database. **(C)** GO analysis of co–expressed genes. **(D)** Co–expressed gene KEGG analysis. *p < 0.05.

To assess the regulatory relationships between the signature genes, we constructed co-expression networks and protein interaction networks. The GSE165004 dataset was used to analyze the co-expression relationships between the signature genes. NCSTN expression was negatively correlated with that of the other six signature genes, while the expression levels of the other six signature genes were positively correlated with one another (**Figure 9A**). To analyze the protein interaction network of the seven signature genes, we created a PPI network using the GeneMANIA database (**Figure 9B**). GO/KEGG analysis was performed on 20 genes to further investigate the function of the signature genes, demonstrating that all 20 co-expressed genes were mainly involved in KEGG signaling pathways (**Figure 9C**), including protein processing in the endoplasmic reticulum, Epstein-Barr virus infection, viral arcinogenesis, bacterial invasion of epithelial cells, the cell cycle and the FoxO signaling pathway (**Figure 9D**).

### Construction and testing of a signature gene-based line graph for predicting RM

**Figure 10:**
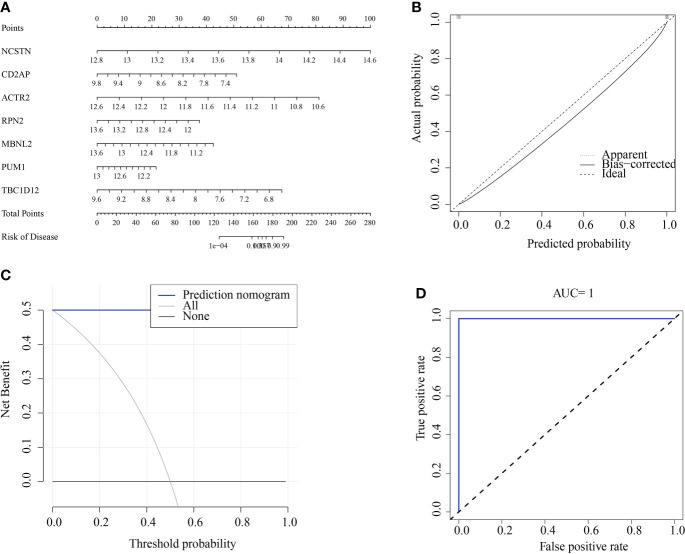
Construction and validation of a RM diagnostic column line graph model. **(A)** Column line graphs were used to predict the occurrence of RM. **(B)** Calibration curves assessed the predictive power of the column line graph model. **(C)** DCA curves were used to assess the clinical value of the column line graph model. **(D)** ROC curves assessed the clinical value of the column line graph model.

We constructed a RM diagnostic column line graph model (**Figure 10A**) using the “Rms” R package for the signature genes (ACTR2, CD2AP, MBNL2, NCSTN, PUM1, RPN2, and TBC1D12), and assessed its predictive power using calibration curves. The calibration curves showed minimal differences between true and predicted RM risk, indicating that the bar graph RM model was very accurate (**Figure 10B**). Decision curve analysis (DCA) suggested that patients could benefit from such nomograms (**Figure 10C**). The correctness of the model was also confirmed using ROC curve analysis (**Figure 10D**).

### Association of signature genes with immune cell infiltration in RM patients

**Figure 11:**
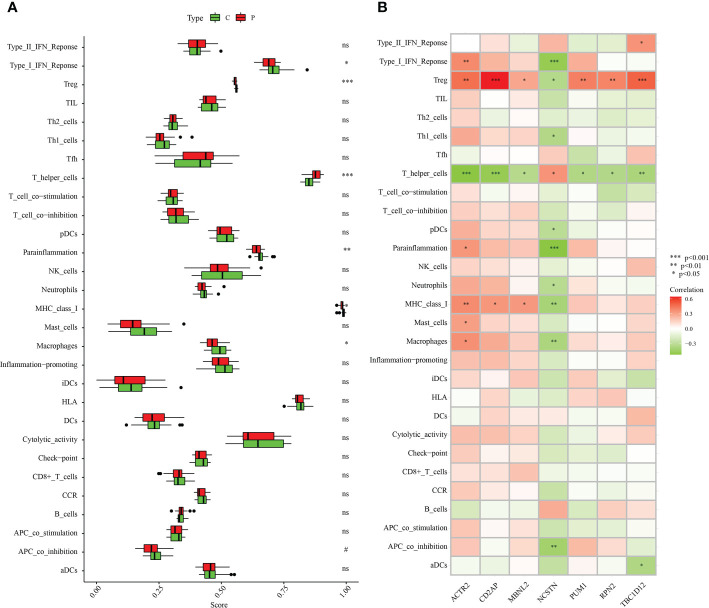
Correlation between the hub genes and immunity. **(A)** Comparison of the ssGSEA scores of the immune cells and immune pathways between the RM group and healthy controls. **(B)** Correlation between characteristic genes and immunity. *p < 0.05, **p < 0.01, ***p < 0.001. 0.05 < p < 0.2. NS, no significance.

The association of immune infiltration between RM patients and healthy controls was further investigated in the GSE165004 dataset using the ssGSEA algorithm. When the results excluded immune cell types that were not statistically significant, Type_I_IFN_Reponse, Treg, Parainflammation, MHC_class_I and Macrophages were significantly down-regulated in RM patients while T_helper_cells were significantly up-regulated in RM patients compared with healthy controls (**Figure 11A**). We then analysed correlations between the signature genes and immune cells and immune-related functions. ACTR2 was significantly negatively correlated with T_helper_cells, but significantly positively correlated with a variety of other immune cells and immune-related pathways (**Figure 11B**). In contrast, NCSTN was significantly positively correlated with T_helper_cells and negatively correlated with multiple other immune cells and immune-related pathways (**Figure 11B**). Treg was significantly negatively correlated with NCSTN and significantly positively correlated with the six other signature genes, T_helper_cells was significantly positively correlated with NCSTN and significantly negatively correlated with the six other signature genes, ACTR2 was significantly positively correlated with macrophages and NCSTN was significantly negatively correlated with macrophages (**Figure 11B**).

### Validation of ACTR2 and NCSTN expression using the TISCH database

**Figure 12:**
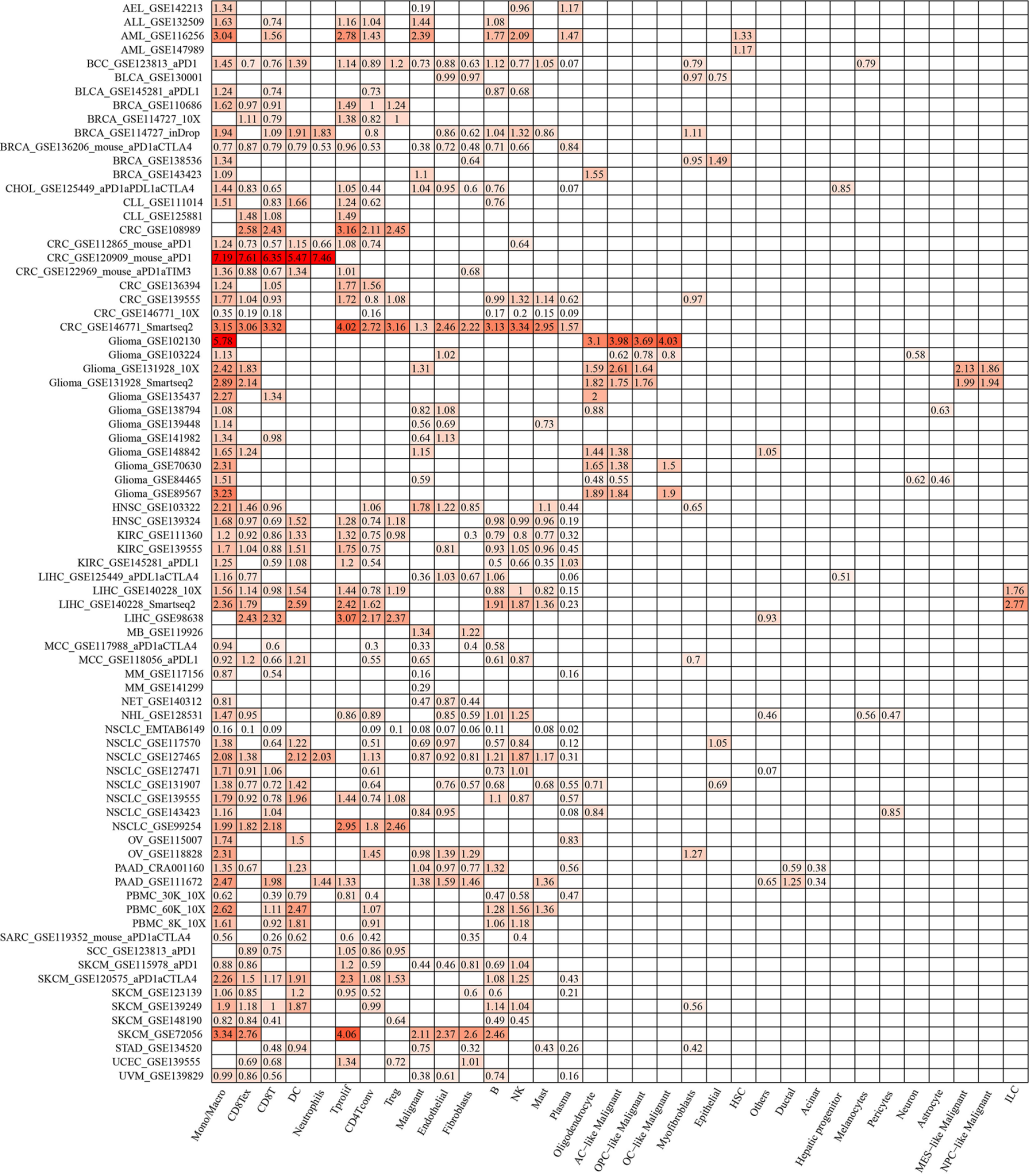
ACTR2 distribution using TISCH scRNA seq database.

**Figure 13:**
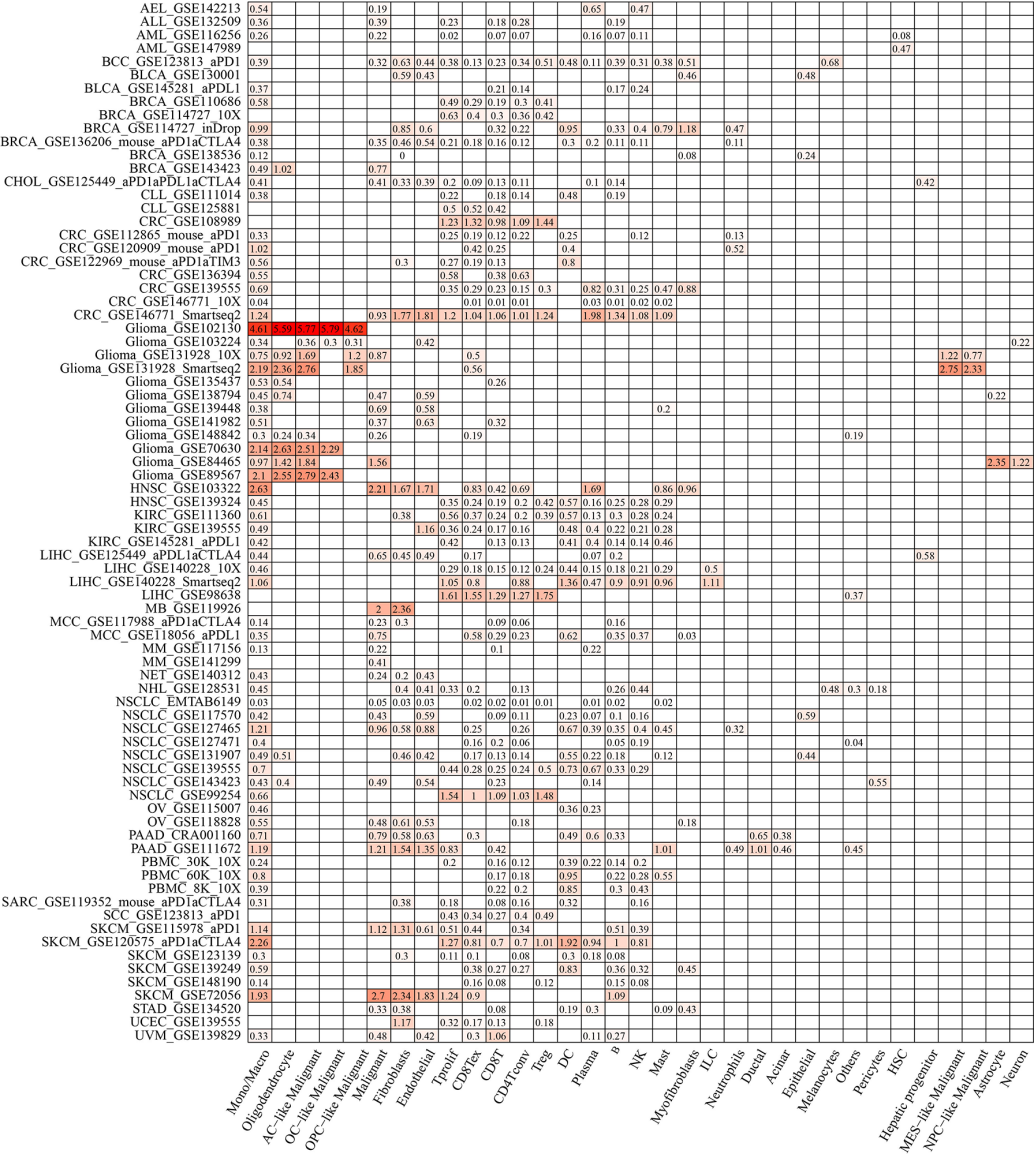
NCSTN distribution using TISCH scRNA seq database.

ACTR2 and NCSTN were significantly associated with immune infiltration in RM patients. When the expression levels of ACTR2 and NCSTN in 33 cell types at the single cell level were analyzed using the TISCH database, it was found that both ACTR2 and NCSTN had the highest expression levels in mono/macro cells across multiple tumor datasets (**Figures 12**, **13**), and had increased expression in mono/macro cells. ACTR2 was expressed in CD8Tex, CD8T, DC, neutrophils, Tproli CD4Tconv, Treg, malignant, endothelial, fibroblasts, B, NK, Mas and Plasma cells (**Figure 12**). Similar to ACTR2, NCSTN had higher expression levels in malignant, fibroblasts, endothelial, Tprolif, CD8Tex, CD8T, CD4Tconv, Treg, DC, Plasma, B and NK cells (**Figure 13**).

### Identification of drug candidates

To facilitate the development of future RM therapies, we performed drug target enrichment analysis using the 7 RM key genes. **Table 3** lists the 17 drug candidates that were evaluated. Irinotecan could target four RM key genes (MBNL2, TBC1D12, PUM1 and CD2AP), while the other six candidates (Alsterpaullone, Camptothecin, Chlorzoxazone, GW-8510, POTASSIUM and (-)- Epigallocatechin) could target 3 different RM key genes. The remaining 10 drug candidates (Verteporfin, Strophanthidin, Uranium, Azacitidine, Etifenin, Staurosporine, Neostigmine, Meclofenoxate, Captopril and Tyrphostin) could target 2 different RM key genes, while ciclosporin, a common drug used to manage RM clinically, could target MBNL2 (p = 0.023).

**Table 3:** Identification of RM drug candidates.

Drug	P-value	Odds Ratio	Combined Score	Drug Targets
**Irinotecan**	4.88E-04	19.68980021	150.1446587	MBNL2;TBC1D12;PUM1;CD2AP
**Alsterpaullone**	0.011055064	9.478342428	42.69866877	MBNL2;TBC1D12;PUM1
**Camptothecin**	0.011584032	9.306841046	41.49108607	MBNL2;TBC1D12;PUM1
**Chlorzoxazone**	0.007629886	10.93725643	53.32658862	ACTR2;RPN2;PUM1
**GW-8510**	0.007696558	10.9009324	53.05464254	MBNL2;TBC1D12;PUM1
**POTASSIUM**	0.022293962	7.162796834	27.24326379	MBNL2;TBC1D12;CD2AP
**(-)-Epigallocatechin**	0.029804748	6.349786932	22.3073575	MBNL2;TBC1D12;CD2AP
**Verteporfin**	0.003997787	28.05978648	154.9465424	NCSTN;RPN2
**Strophanthidin**	0.005850578	22.98362573	118.1637568	ACTR2;PUM1
**Uranium**	0.002989614	32.64628099	189.7601315	ACTR2;PUM1
**Azacitidine**	0.016801262	13.08600337	53.47335226	TBC1D12;PUM1
**Etifenin**	0.017560467	12.77495881	51.63772641	ACTR2;PUM1
**Staurosporine**	0.019811159	11.96043277	46.90295556	TBC1D12;PUM1
**Neostigmine**	0.019868958	11.94135802	46.79336577	ACTR2;PUM1
**Meclofenoxate**	0.023657885	10.84781997	40.61487591	ACTR2;NCSTN
**Captopril**	0.033293867	8.96440281	30.5003233	ACTR2;PUM1
**Tyrphostin**	0.010353604	16.98521739	77.62958757	TBC1D12;PUM1

## Discussion

Recurrent miscarriage (RM) is a common reproductive complication that affects 1-3% of women during their reproductive years (42). Substantial progress has recently been made in the study of pathologic factors associated with RM. Immune factors have been of particular interest. A large number of studies have reported an association between immune cells and RM, in particular the role of macrophages. Macrophages account for 20–25% of all metaphase leukocytes at the implantation site and play an important role in regulating the maternal immune microenvironment by linking the adaptive and innate immune systems (14). Abnormal macrophage infiltration at the maternal-fetal interface has been associated with RM (43–45). Macrophages polarize into M1/M2 subtypes under specific circumstances; M1 macrophages produce pro-inflammatory cytokines and regulate the inflammatory response while M2 macrophages promote tissue remodeling and repair (29). Early reports suggest that metaphase macrophages (dMΦ) may polarize into the M2 subtype during a normal pregnancy (18), In contrast, macrophages during complicated pregnancies, such as RM or pre-eclampsia, polarize into the M1 subtype and mediate the inflammatory response (19, 44).

The rapid development of scRNA-seq technology has allowed researchers to explore the molecular characteristics of tumor-infiltrating immune cells in tumor microenvironment (TME). However, most works to this end have focused on adaptive immune cells. The role of innate immune cells has not received sufficient attention, which may significantly affect patient prognosis and response to treatment, especially with respect to immunotherapy. Pan et al. used single cell sequencing analysis to identify severe disruption of dNK cell polarization in the setting of an unexplained recurrent spontaneous abortion (URSA), whereas dNK cells normally interact with extravillous trophoblasts to achieve immune tolerance polarization (29). Zhu et al. used single-cell sequencing analysis to find proportionate differences in NK cell and macrophage metaphases in patients with recurrent spontaneous abortion (RSA) compared with normal controls (46). Du et al. found that stromal cells were the most abundant cell type in the meconium during early pregnancy and that communication between stromal cells and other cell types, such as over-activation of macrophages and NK cells, was significantly impaired in patients with recurrent spontaneous abortion (RSA) (47).

This study screened 186 macrophage marker genes associated with recurrent miscarriage from the GSE165004 dataset, of which 90 were significantly upregulated in the disease group and 96 were significantly downregulated in the disease group (**Figures 2A, B**). Subsequent GO enrichment analysis revealed that all DEGs were enriched in signaling pathways such as the negative regulation of protein phosphorylation, type I interferon signaling pathway, and defense response to viruses (**Figure 2C**), while KEGG enrichment analysis showed that DEG was mainly involved in endocytosis, human cytomegalovirus infection, the Apelin signaling pathway, and Th17 cell differentiation (**Figure 2D**). These pathways may therefore be closely related to the progression of RM.

This study used three machine learning algorithms (LASSO, SVM-RFE, and Random forest) to identify seven macrophage marker genes (ACTR2, CD2AP, MBNL2, NCSTN, PUM1, RPN2, and TBC1D12) that were associated with RM in the GSE165004 dataset. Most of these marker genes are associated with progression or macrophage activity in patients with RM. ACTR2, also known as actin-related protein 2, has been previously implicated in the development of RM and is closely associated with the development of lung cancer (48), liver cancer (49) and primary thrombocythemia (50). CD2-associated protein (CD2AP), a scaffolding protein that controls actin dynamics, is closely associated with the development of Alzheimer’s disease (51)、gastric cancer (52) and acute promyelocytic leukemia (53). MBNL2 belongs to a family of highly conserved RNA-binding proteins that drive cancer cell proliferation and migration by regulating the expression of hypoxia-inducible genes, such as VEGF-A, and variable splicing (54); MBNL2 expression levels were significantly negatively correlated with hepatocellular carcinoma tumor size and stage, and MBNL2 overexpression *in vitro* and *in vivo* inhibited the growth and invasion of hepatocellular carcinoma cells (55); MBNL2 also regulates tumor cell proliferation and the DNA damage response by stabilizing p21 (56). Nicastrin (NCSTN) is the core subunit of γ-secretase and is essential to the intracellular transport and stability of γ-secretase and the recognition of γ-secretase substrates (57). NCSTN is significantly upregulated in breast cancer and induces epithelial-mesenchymal transition (EMT) through Notch1 cleavage (58). NCSTN was also the response of colon cancer to chemotherapy (59). PUM1 is also an RNA-binding protein that regulates gene expression by binding to the mRNA of target genes. Previous studies have shown that PUM1 expression is elevated in pre-eclampsia and is associated with the proliferation and migration of trophoblast cells (60). RPN2 is an important component of the oligosaccharyltransferase complex and is responsible for the N-glycosylation of many proteins (61). RPN2 was found to promote the malignant progression of breast cancer (62), gastric cancer (63) and colon cancer (64). RPN2 promotes docetaxel resistance in breast cancer cells by mediating CD63 glycosylation (65). TBC1D12 is a Rab11-binding protein that regulates neuroectodermal growth in PC12 cells (66). These prior associations further support that the macrophage marker genes associated with RM identified in this study may provide potential targets for laboratory experimental design to elucidate the molecular mechanisms underlying RM progression.

To further illustrate the role of central genes in RM, we performed GSEA analysis, which showed that the complement and coagulation cascades were significantly enriched in the highly expressed subgroups of all of the target genes except for NCSTN and PUM1. DNA replication was significantly enriched in the highly expressed subgroups of ACTR2, CD2AP, PUM1 and RPN2, and Hedgehog signaling pathway was significantly enriched in the highly expressed subgroups of ACTR2, CD2AP, and PUM1. Subsequent ROC analysis showed that all of the central genes had potential diagnostic value in the clinical management of RM. To further demonstrate the clinical diagnostic value of these 7 genes, we collected 5 normal samples and 5 recurrent miscarriage samples for validation. The expression pattern of these 7 RM genes was consistent with that of GSE165004, which further supports the diagnostic properties of the 7 core genes for RM. To further elucidate the regulatory mechanisms of the 7 central genes, we analysed the co-expression profiles and interacting proteins of the seven central genes. Positive co-expression patterns existed between all genes, except for NCSTN, which was negatively co-expressed with all of the other genes. We then determined that the seven central genes enriched the function of 20 interacting proteins that were mainly involved in the Notch signaling pathway, the response to antibiotics and protein processing in the endoplasmic reticulum. Finally, we determined that ACTR2 was positively associated with several of the 50 HALLMARK signaling pathways, while NCSTN was negatively associated with several pathways. These findings suggest that ACTR2 may regulate RM progression mainly through signaling pathway activation, while NCSTN affects RM progression through pathway inhibition.

Since the seven core genes we identified belong to macrophage marker genes, we speculated that they regulate the progression of RM by modulating immune cell infiltration. Macrophage infiltration was significantly lower in RM than in normal samples. Correlation analysis showed that ACTR2 was significantly positively correlated with macrophage infiltration while NCSTN was significantly negatively correlated with macrophage infiltration. When we analysed the expression patterns of ACTR2 and NCSTN in different immune cells in the TISCH database, we found that both ACTR2 and NCSTN were highly expressed in macrophages/monocytes. Taken together, these results suggest that ACTR2 and NCSTN may influence the progression of RM by regulating macrophage infiltration.

In order to induce the overall immune tolerance of the mother to the fetus and maintain immune reactivity to all other foreign antigens, the maternal immune system needs to be adjusted. These changes involve different immune cells and cytokines. This process, in addition to immunomodulation, also involves the destruction of uterine tissue, vascular remodeling and placental formation to jointly ensure the establishment, maintenance, development and termination of normal pregnancy (67). Many studies have shown that unexplained recurrent abortions are related to immune factors. Immune-related abortion can be divided into autoimmune RM and alloimmune RM. The former is primarily seen in the setting of antiphospholipid syndrome, while the latter is the result of abnormalities in the number and function of immune cells. Macrophages, which account for 20-30% of all leukocytes in the decidua, can promote endometrial receptivity, uterine spiral artery remodeling and trophoblast invasion by secreting a variety of cytokines, including Th1 cytokines secreted by M1 macrophages and Th2 cytokines secreted by M2 macrophages. The expression balance of Th1/Th2 plays an important role in inducing the maternal immune system to tolerate the fetus (68). Immune imbalance at the maternal fetal interface can lead to immune system activation and an inflammatory reaction. Inflammatory signals induce the expression of relevant tissue factors such as endothelial cells and monocytes, which further trigger the procoagulant response and produce a systemic hypercoagulable state. From the perspective of potential genetic factors and epigenetics, polymorphisms and variations in genes regulating coagulation function and immune function may be potential contributors to RM (69, 70). Anticoagulation and immunotherapy have been shown to improve the reproductive outcome of patients with cellular immune abnormalities and thrombosis (71), and aspirin and heparin are widely used to reduce the risk of recurrent abortion in women with antiphospholipid syndrome (72). Recent studies have shown that low-dose aspirin can inhibit excessive or persistent inflammation at the maternal fetal interface of RM patients by reducing the level of HMGB1 protein expressed by the serum and decidual macrophages (73), Some immunosuppressants, such as cyclosporine A, can achieve a therapeutic effect by regulating interactions between immune cells and inducing Th2 type immune bias at the maternal fetal interface, which are conducive to pregnancy maintenance (74, 75). In order to determine the potential of macrophages as drug targets related to the treatment of recurrent abortions, we screened the DSigDB database for clinical agents that specifically target 7 key RM genes. These 7 candidates targeted 3 different key RM genes, of which Alsterpaullone is a commonly used clinical agent for the treatment of RM. A previous analysis revealed that ACTR2 was significantly associated with macrophage infiltration in RM and could be a key molecular target for future RM drug treatments. We also found that ciclosporin, a key drug in the clinical treatment of RM, can target MBNL2, providing a hint as to its molecular role in the treatment of RM.

In conclusion, we identified and validated seven signature genes using macrophage marker genes that can be used as diagnostic markers for RM. Of these, ACTR2 was significantly positively correlated with macrophage infiltration in RM, while NCSTN was significantly negatively correlated with macrophage infiltration in RM. The interrelationship between these candidate genes and may influence RM progression through macrophage regulation.

## Data availability statement

The datasets presented in this study can be found in online repositories. The name of the repository and accession number can be found below: Gene Expression Omnibus, GSE214607.

## Ethics statement

The studies involving human participants were reviewed and approved by Medical Ethics Committee of the First Affiliated Hospital of Guangxi Medical University. The patients/participants provided their written informed consent to participate in this study.

## Author contributions

YY and XF supervised the project and designed the study. PW and MD wrote the manuscript and prepared the figures and tables. BL, TL and WH edited the manuscript. YB and SC performed the research. All authors contributed to the final manuscript and approved the submitted version. All authors listed made substantial, direct and intellectual contributions to this work and were approved for publication.

## Funding

This research was financially supported by grants to YY from the Natural Science Foundation of Guangxi Zhuang Autonomous Region (2019GXNSFFA245013, 2018GXNSFDA050017); the National Natural Science Foundation of China (NO. 81871172); and the Guangxi Medical University Training Program for Distinguished Young Scholars. This research was also supported by Special Fund of the Female Fertility Preservation Innovation Team of the First Affiliated Hospital of Guangxi Medical University).

## Acknowledgments

All authors thank the sample providers and research teams of the GSE165004 and GSE26787 cohorts who provided data for this article.

## Conflict of interest

The authors declare that the research was conducted in the absence of any commercial or financial relationships that could be construed as a potential conflict of interest.

## Publisher’s note

All claims expressed in this article are solely those of the authors and do not necessarily represent those of their affiliated organizations, or those of the publisher, the editors and the reviewers. Any product that may be evaluated in this article, or claim that may be made by its manufacturer, is not guaranteed or endorsed by the publisher.
